# Self-reported health status and mortality from all-causes of death, cardiovascular disease and cancer in an older adult population in Spain

**DOI:** 10.1371/journal.pone.0261782

**Published:** 2022-01-21

**Authors:** Laura Torres-Collado, Manuela García de la Hera, Laura María Compañ-Gabucio, Alejandro Oncina-Cánovas, Sandra González-Palacios, Leyre Notario-Barandiaran, Jesús Vioque

**Affiliations:** 1 Instituto de Investigación Sanitaria y Biomédica de Alicante, ISABIAL-UMH, Alicante, Spain; 2 Unidad de Epidemiología de la Nutrición, Departamento de Salud Pública, Historia de la Ciencia y Ginecología, Universidad Miguel Hernández (UMH), Alicante, Spain; 3 CIBER Epidemiología y Salud Pública (CIBERESP), Instituto de Salud Carlos III, Madrid, Spain; Institut d’Investigacio Biomedica de Bellvitge, SPAIN

## Abstract

**Aim:**

To assess the association between self-reported health (SRH) and mortality from all-causes, cardiovascular disease (CVD) and cancer, in adults 65 years and older in Spain.

**Methods:**

We analysed data of 894 adults (504 women, 390 men) aged 65 years and above from two population-based studies, the EUREYE-Spain study and the Valencia Nutritional Survey (VNS). SRH was assessed at baseline using a single question which is widely used in epidemiological studies: “Overall, how would you consider your health at present?” and the response options were: 1. Very good, 2. Good, 3. Fair, 4. Poor, 5. Very poor. Deaths were ascertained during a 12-year follow-up period, and we used Cox proportional hazards regression models to obtain adjusted hazard ratios (HR).

**Results:**

During the 12 years of follow-up (8566.2 person-years), we observed 400 deaths, 158 (39.5%) due to CVD and 89 (22.3%) due to cancer. Fair and poor/very poor SRH were significantly associated with higher all-cause mortality after 12-years of follow-up, HR = 1.29 (95% CI, 1.03–1.61) and HR 1.53 (95% CI, 1.09–2.15), respectively. We observed evidence of higher CVD mortality among those who reported fair and poor/very poor SRH, although the association was attenuated and lost statistical significance in the fully adjusted models.

**Conclusion:**

This study suggests that a poor SRH status is associated with a higher all-cause mortality risk among older adults in Spain. Checking SHR status may be useful to plan health care in older adults.

## Introduction

Self-reported health (SRH) has been described as a subjective indicator of health status that involves a holistic concept related to physical, mental, and social functioning [1]. This estimator is widely used in epidemiological studies as it is easy, low-cost and a validated measure in general and older populations [2,3]. Moreover, SRH has been shown to be a good predictor, not only for functional decline and chronic disease risk, but also for mortality risk [4].

The present evidence shows the growing use of SRH as an indicator of all-cause mortality. An early review by Idler et al found a strong association between SRH and mortality from all causes [5], reporting relative risk between 1.5 and 3.0 for poor or fair health perception compared to a good or excellent perceived health status. A subsequent meta-analysis carried out by DeSalvo et al, found that people with poor SRH had a two-fold higher mortality risk compared to those reporting excellent SRH [6]. In addition, studies from Europe have reported a similar inverse association between SRH and all-cause mortality in adults or in older populations [2,7,8]. In Spain, Rodriguez-García showed that older adults who reported poor SRH had 32% higher mortality risk than those reporting a good SRH after a 15-year follow-up study [9]. However, other studies exploring the association between SRH and mortality in adult populations in Spain have found that SRH status could differ depending on sex, socioeconomic status, education level, chronic diseases and demographic characteristics [8,10].

Although most studies described the relationship between SRH and all-cause mortality, few studies focused on cardiovascular disease (CVD) and cancer mortality [10,11]. In this sense, some studies have suggested that the relationship between SRH and mortality could be different according to the cause of death [10,12]. In fact, the study by Fernández-Ruiz et al. showed that SRH was a more significant predictor for stroke and respiratory disease mortality than for diseases such as cancer [10]. Moreover, previous studies seem to support that predictive value of SRH could be poorer in the long-term than the short-term in older adults. Thus, the aim of this study was to assess the association between SRH status and mortality by all-cause, CVD and cancer in an older adult population of 65 years and above in Spain.

## Material and methods

### Study design and population

The study population consisted of 894 participants (504 women and 390 men), aged 65 years and above, who participated in two population-based surveys in the Valencian Region (Spain): The Valencian Nutrition Survey (VNS) and the EUREYE-Spain study. Briefly, the VNS was a nutritional survey based on a representative sample of an adult population aged 15 years and above, carried out in all three provinces of the Valencian Region in 1994 that included 303 participants, aged 65 years and above. The EUREYE study was a multicentre, cross-sectional study which was also based on a representative population aged 65 years and above from seven European countries in 2000–2001, which enrolled 591 participants in the province of Alicante in the Valencian Region, Spain. Details of both studies have been previously published [13–15].

In both studies, participants were interviewed at baseline by trained fieldworkers who used structured questionnaires with questions on sociodemographic characteristics and main lifestyle habits. A health examination including height and weight measurements was also conducted at baseline. All participants provided written informed consent, and ethical approval for the studies was provided by the Local Ethical Committee of the Hospital of San Juan and the University Miguel Hernandez, Alicante, Spain (projects FIS 00/0985 and V FP-EU, QLK6-CT-1999-02094).

### Assessment of self-reported health and other variables

Information on SRH status was collected using a five-point ordinal single question which is widely used in epidemiological studies: “Overall, how would you consider your health at present?” [8,10]. The response options were: 1. Very good, 2. Good, 3. Fair, 4. Poor, 5. Very poor. Since very few respondents reported “very good or very poor” health, we combined the categories, creating a three-category SRH variable: 1. Very good/good, 2. Fair and 3. Poor/very poor.

The study participants also provided information on sociodemographic, lifestyle habits variables at baseline. These variables included: sex (men; women); age (65–74 years; ≥75years); educational level (<primary school; ≥ primary school); measured weight and height that allowed us to estimate and categorize body mass index (BMI) as weight in kilograms divided by the square of height in meters (<25 kg/m2, 25–29 kg/m2, ≥30 kg/m2); measured waist circumference in cm (normal:78–94 cm in men and 64–80 cm in women; moderate risk: 94–102 cm in men and 80–88 cm in women; and high risk: >102 cm in men and >88 cm in women); smoking (never; former; current); physical activity in leisure time (very low; moderately active-high); television watching (hours/day); hours of sleep (hours/day); relative Mediterranean Diet score (rMED), a dietary quality index with a range of 0–18 points, derived from a food frequency questionnaire validated in a Spanish older adult population [16]. We also collected the presence of chronic disease such as self-reported hypertension (no/yes), diabetes (no/yes) and high blood cholesterol (no/yes) at baseline. Previous studies have shown a high level of concordance between self-reported diseases and those documented in medical records in adult populations [17,18].

### Assessment of mortality

Information on the cause and date of death during the 12-year follow-up period was collected through the Mortality Registry in the Valencian Region and the National Death Index from the Spanish Statistical Office. The cause of death was codified according to version 10 of the International Classification of Diseases (ICD-10). For analysis, we combined deaths in three broad categories as follows: cardiovascular disease (ICD-10: I00-I99), cancer (ICD-10 codes: C00-D49), and all-cause mortality, which included the two first categories as well as deaths from any other cause.

### Statistical analysis

We used chi-square and analysis of variance (ANOVA) tests to compare the baseline differences by SRH categories. Participants were reclassified in three categories of SRH status as very good/good, fair and poor/very poor. For each participant we calculated the person-years of follow-up from the date of baseline interview in each study to the date of death or completion of the 6- and 12-year follow-up, whichever came first. In addition, we examined the association between SRH and risk of mortality at 6 and 12 years of follow-up.

The presence of heterogeneity between the two studies was evaluated using I^2^ and Cochran’s test [19]. As the results between these studies did not show heterogeneity, the results were combined, adjusting for a potential study effect using a dichotomous variable (VNS/EUREYE study).

We used Cox proportional regression to estimate adjusted hazard ratios (HR) and 95% confidence intervals (95% CI) for each category of SRH in comparison to the lower categories (very good/good, fair, poor/very poor). We show two models in the tables: one adjusted for age and sex; a second more fully adjusted model, adding variables that have been reported previously as potential confounders in the literature and those variables showing p-values and <0.20 in bivariate analysis. In addition, the non-zero slope of the scaled Schoenfeld residuals on the time function showed that the proportional hazard assumption was met. We used Likelihood Ratio Test (LRT) to assess the overall significance of the association using SRH as categorical variable in each model explored. Finally, since age, sex and several diseases have been shown to be associated with mortality, we carried out several sensitivity analyses: a) stratifying by sex b) stratifying by age; c) excluding patients with history of stroke or cardiovascular disease; d) including only patients with history of hypertension, e) including only patients with history of diabetes and f) including only patients with history of cholesterol. In sensitivity analyses, models were adjusted for the same variables used in the fully adjusted models without including the variable used for stratification in each sensitivity analysis.

All analyses were performed using STATA, version 16® College Station, TX: StataCorp LP. The applied statistical tests were bilateral, and significance was established at 0.05.

## Results

**Table 1** shows the general characteristics of the participants according to categories of SRH at baseline. Those who reported poor/very poor SRH status presented a higher proportion of women, lower educational level, never smokers, very low physical activity in leisure time and hypertension. Participants reporting poor/very poor SRH status also showed a lower proportion of self-reported diabetes and cholesterol.

**Table 1:** Socio-demographic and lifestyle characteristics according to self-reported health among elderly participants (65 years and above) of the EUREYE-Spain and the Valencia Nutrition studies in Spain (n = 894).

		Self-reported health			
	Total	Very good/good	Fair	Poor/very poor	P-value^1^
Study, n (%)	894	500 (55.9)	309 (34.6)	85 (9.5)	0.001
EUREYE-Spain	591 (66.1)	356 (71.2)	182 (58.9)	53 (62.3)	
VNS	303 (33.9)	144 (28.8)	127 (41.1)	32 (37.7)	
Sex, n (%)					<0.001
Women	504 (56.4)	250 (50.0)	198 (64.1)	56 (65.9)	
Men	390 (43.6)	250 (50.0)	111 (35.9)	29 (34.1)	
Age, n (%)					0.29
65–74 years	560 (62.6)	323 (64.6)	189 (61.2)	48 (56.5)	
≥ 75 years	334 (37.4)	177 (35.4)	120 (38.8)	37 (43.5)	
Education Level, n (%)					<0.001
< Primary school	584 (65.3)	294 (58.8)	228 (73.8)	62 (72.9)	
≥ Primary school	310 (34.7)	206 (41.2)	81 (26.2)	23 (26.1)	
Body Mass Index (Kg/m^2^), n (%)					0.40
<25 kg/m^2^	167 (18.8)	93 (18.6)	56 (18.4)	18 (21.4)	
25–29 kg/m^2^	412 (46.4)	241 (48.2)	130 (42.7)	41 (48.8)	
≥30 kg/m^2^	309 (34.8)	166 (33.2)	118 (38.8)	25 (29.8)	
Waist circumference^2^, n (%)					0.33
Normal	97 (11.0)	59 (11.9)	32 (10.4)	6 (7.4)	
Moderate risk	203 (23.0)	122 (24.7)	66 (21.5)	15 (18.5)	
High risk	582 (66.0)	313 (63.4)	209 (68.1)	60 (74.1)	
Smoking Status, n (%)					0.02
Never	567 (63.6)	296 (59.3)	214 (69.5)	57 (67.1)	
Former	205 (23.0)	122 (24.5)	63 (20.4)	20 (23.5)	
Current	120 (13.4)	81 (16.2)	31 (10.1)	8 (9.4)	
Diabetes^3^ (yes), n (%)	174 (19.5)	83 (16.6)	66 (21.4)	25 (29.4)	0.01
Cholesterol^3^ (yes), n (%)	180 (20.3)	85 (17.1)	73 (23.9)	22 (25.9)	0.03
Hypertension^3^ (yes), (n %)	355 (40.1)	171 (34.5)	140 (45.7)	44 (52.4)	<0.001
Physical activity at leisure time, n (%)					<0.001
Very low	499 (56.5)	245 (49.3)	195 (64.1)	59 (71.9)	
Moderately active and high	384 (43.5)	252 (50.7)	109 (35.9)	23 (28.1)	
TV-watching, hours/day (mean SD)	3.8 (1.9)	3.7 (1.9)	3.9 (1.9)	3.8 (2.5)	0.65
Sleeping time, hours/day (mean SD)	7.8 (2.0)	7.9 (1.8)	7.7 (2.1)	7.6 (2.7)	0.34
rMED, mean (SD)	8.2 (2.4)	8.3 (2.5)	8.2 (2.4)	8.1 (2.2)	0.29

Abbreviations: SD, Standard deviation; VNS, Valencia Nutrition Survey; EUREYE-Spain Survey; BMI, Body Mass Index; rMED, relative Mediterranean Dietary index.

^1^ P-value from chi-square test (categorical variables) and ANOVA (continuous variables).

^2^ Waist circumference: Normal (78–94 cm in men and 64–80 cm in women), moderate risk (94–102 cm in men and 80–88 cm in women), high risk (>102 cm in men and >88 cm in women).

^3^ Self-reported diabetes (no/yes), high cholesterol (no/yes) and hypertension (no/yes).

**Fig 1:**
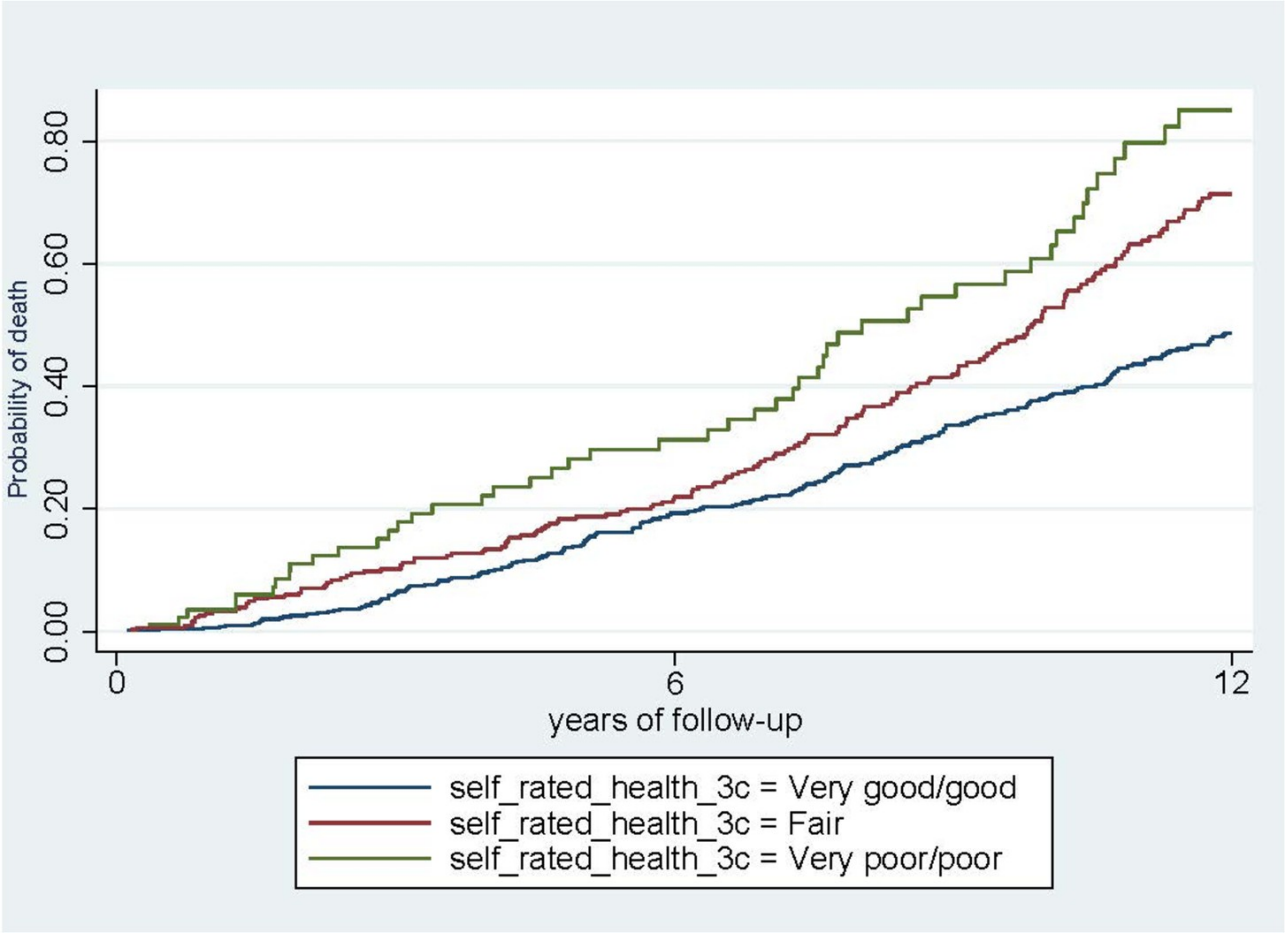
Curves of cumulative incidence for all-cause mortality during the study period according to SRH among elderly participants of EUREYE-Spain study and Valencia Nutrition Survey in Spain (n = 894).

**Table 2** displays crude and adjusted models for mortality at 6 and 12-year follow-up according to SRH categories at baseline. At 6 years of follow-up (4908.4 person-years), we observed 172 deaths, 67 (39%) from CVD and 44 (26%) from cancer and at the end of the 12 years of follow-up (8566.2 person-years), we observed 400 deaths, 158 (39.5%) due to CVD and 89 (22.3%) due to cancer. Fig 1 presents the cumulative incidence for all-cause mortality during the 12 years of follow-up according to SRH categories. In general, participants with poor/very poor SRH showed curves with higher incidence of deaths.

**Table 2:** Associations between self-reported health and all-cause, cardiovascular disease and cancer mortality among elderly participants of EUREYE-Spain study and Valencia Nutrition Survey in Spain (n = 894).

	Self- reported health				
	Very good/good	Fair	Poor/very poor	p-value^2^	
	*Follow-up at 6 years*				
All-cause (n, %)	500 (55.9)	309 (34.6)	85 (9.5)		
deaths, n	88	61	23		
person-years	2798.5	1673.7	436.2		
HR (95% CI)					
Age and sex adjusted	1.00	1.21 (0.87–1.69)	1.73 (1.09–2.74)	0.07	
Fully adjusted model^1^	1.00	1.03 (0.73–1.47)	1.34 (0.79–2.25)	0.57	
CVD (n, %)	444 (56.2)	273 (34.6)	72 (9.3)		
deaths, n	32	25	10		
person-years	2586.8	1570.3	395.7		
HR (95% CI)					
Age and sex adjusted	1.00	1.23 (0.72–2.08)	1.92 (0.94–3.94)	0.22	
Fully adjusted model^1^	1.00	0.93 (0.53–1.65)	1.28 (0.56–2.92)	0.77	
Cancer (n, %)	438 (57.2)	263 (34.3)	65 (8.5)		
deaths, n	26	15	3		
person-years	2568.7	1522.8	385.1		
HR (95% CI)					
Age and sex adjusted	1.00	1.03 (0.54–1.95)	0.80 (0.24–2.65)	0.92	
Fully adjusted model^1^	1.00	1.13 (0.57–2.24)	0.99 (0.28–3.41)	0.93	
	*Follow-up at 12 years*				
All-cause (n, %)	500 (55.9)	309 (34.6)	185 (9.5)		
deaths, n	193	158	49		
person-years	4964.9	2873.2	728.1		
HR (95% CI)					
Age and sex adjusted	1.00	1.51 (1.22–1.87)	1.81 (1.32–2.49)	<0.001	
Fully adjusted model^1^	1.00	1.29 (1.03–1.61)	1.53 (1.09–2.15)	0.02	
CVD (n, %)	383(58.7)	212 (32.5)	57 (8.7)		
deaths, n	76	61	21		
person-years	4196.2	2227.9	557.2		
HR (95% CI)					
Age and sex adjusted	1.00	1.54 (1.09–2.17)	1.93 (1.19–3.14)	0.007	
Fully adjusted model^1^	1.00	1.19 (0.83–1.71)	1.45 (0.84–2.50)	0.35	
Cancer (n, %)	359 (61.6)	181 (31.0)	43 (7.4)		
deaths, n	52	30	7		
person-years	4002.5	1978.5	479.0		
HR (95% CI)					
Age and sex adjusted	1.00	1.35 (0.85–2.13)	1.19 (0.54–2.63)	0.44	
Fully adjusted model^1^	1.00	1.33 (0.82–2.87)	1.25 (0.54–2.87)	0.49	

Abbreviations: CI: Confidence interval; CVD: Cardiovascular disease.

^1^ Cox proportional hazard ratios adjusted for age (65–74 years; ≥75 years), sex, study (EUREYE study, Valencia Nutrition Survey), educational level (<Primary, ≥Primary), BMI (<25, 25.0–29.9, ≥ 30), waist circumference (normal, moderate and high risk), sleeping time (hours/day), smoking habit (current; past and never), self-reported diabetes (no/yes), high cholesterol (no/yes), hypertension (no/yes), relative Mediterranean Diet (as continuous term),main physical activity at leisure time (low, moderate-high) and TV-watching (hours/day).

^2^p-value from likelihood ratio test.

We observed a higher risk of all-cause mortality among participants with fair and poor/very poor SRH. Compared to participants who reported very good/good SRH, those participants in the higher category (poor/very poor) showed higher risk of all-cause mortality after 6 years of follow-up. The age and sex adjusted HR was 1.73 (95% CI 1.09–2.74), although the association was attenuated after adjusting for potential confounders 1.34 (95% CI 0.79–2.25). At 12-years of follow-up, participants who reported having fair SRH or poor/very poor SRH, showed higher all-cause mortality compared to those with very good/good SRH in the multivariable model, HR 1.29 (95% CI 1.03–1.61) and HR 1.53 (1.09–2.15) respectively. In addition, higher CVD mortality was observed among those who reported fair SRH, HR 1.54 (95% CI 1.09–2.17) and poor/very poor SRH, HR 1.93 (95% CI 1.19–3.14) in the age and sex-adjusted model although the association lost significance in the more-fully adjusted models. No significant association was observed for cancer mortality.

**Table 3** shows the sensitivity analyses of the association between SRH and mortality. The association between poor/very poor SRH status and mortality was stronger among VNS participants (HR = 2.56) than the observed in the EUREYE participants (HR = 1.23), although when we explored an interaction between SRH status and study (EUREYE/VNS), it was not statistically significant (p = 0.26). When we only included women in the analysis, we observed that compared to participants with very good/good SRH, participants with fair and poor/very poor SRH showed higher risk of all-cause mortality, HR 1.40 (95% CI 1.00–1.96) and HR 1.65 (95% CI 1.03–2.65), respectively. Finally, we observed a higher all-cause mortality among participants of less than 75 years old who reported fair SRH, HR 1.75 (95% CI 1.24–2.46) and those who described poor/very poor SRH, HR 2.12 (95% CI 1.25–3.60).

**Table 3:** Hazards ratios (HR) resulting from sensitivity analyses between self-reported health and all-cause mortality at 12 years of follow-up among elderly participants of EUREYE-Spain study and Valencia Nutrition Survey in Spain (n = 894).

	Self-reported health								
	n	Deaths	Very good/good (reference)		Fair^1^		Poor/very poor^1^		p-value^2^
Sensitivity analysis including only:			n		n		n		
Participants from EUREYE study	591	250	356	1.00	182	1.28 (0.97–0.69)	53	1.23 (0.79–1.92)	0.21
Participants from VNS study	303	150	144	1.00	127	1.44 (0.96–2.13)	32	2.56 (1.39–4.72)	0.01
Men	390	206	250	1.00	111	0.99 (0.60–1.61)	29	1.39 (0.66–2.93)	0.68
Women	504	194	250	1.00	198	1.40 (1.00–1.96)	56	1.65 (1.03–2.65)	0.05
Participants <75years	560	176	323	1.00	189	1.75 (1.24–2.46)	48	2.12 (1.25–3.60)	0.001
Participants ≥75 years	334	224	177	1.00	120	1.05 (0.77–1.42)	37	1.21 (0.76–1.93)	0.72
Participants without CD	362	115	242	1.00	99	1.23 (0.64–2.35)	21	0.95 (0.29–3.08)	0.82
Participants with hypertension	355	173	171	1.00	140	1.01 (0.59–1.71)	44	0.88 (0.39–1.99)	0.94
Participants with diabetes	174	110	83	1.00	66	1.26 (0.80–1.98)	25	1.38 (0.73–2.63)	0.47
Participants with high cholesterol	180	70	85	1.00	73	1.27 (0.70–2.32)	22	1.09 (0.48–2.49)	0.73

^1^ Values are HR estimated with Cox regression and 95% CI. HR were adjusted for the same variables as in Table 2 without including the variable used in each sensitivity analysis.

^2^ p-value from likelihood ratio test.

## Discussion

In the present study, conducted in an older adult population aged 65 years and above, we found that fair and poor/very poor SRH was significantly associated with a higher all-cause mortality after 12-years of follow-up.

The effect of SRH on several health outcomes has been the object of research in recent years, especially regarding all-cause mortality, although there is some evidence that the association between SRH and mortality can vary according to specific causes of death [3,10]. With regard to all-cause mortality, the positive associations observed in our study between the less favourable SRH status and all-cause mortality are consistent with previous literature [2,10,20]. At 6-years of follow-up, we observed an almost significant association between SRH and mortality in the age and sex model adjusted. We found that those who reported poor/very poor SRH had 73% higher all-cause mortality. We also observed a 29% and 53% significant higher risk of all-cause mortality among those with fair and poor/very poor SRH at 12-years of follow up, respectively, which are in line with previous studies. A Finnish study carried out with community-dwelling adults aged 70 and above with a 27-year follow-up period observed that SRH was a stronger predictor of all-cause mortality during the first few years, and that the association was gradually attenuated towards the end of the follow-up [2]. Previous studies have suggested that this attenuation may be related to the limitations of SRH when predicting long-term mortality risk at the end of life, when the presence of comorbidity and frailty status are common [2,21–23]. However, a prospective cohort study has shown that SRH can predict mortality in an older population aged 75 and above, despite the fact that it decreases with advancing age [24].

Regarding the relationship between SRH and cause-specific mortality, several studies have shown inconsistent results, probably due to differences in the type of population, study design, the way SRH was classified and the mortality evaluated [8,12,20]. In relation to CVD mortality, the Neurological Disorders in Central Spain study (NEDICES), a longitudinal population-based survey which includes people aged 65 years and older showed an almost statistically significant association between poor/very poor SRH and mortality due to CVD (HR 1.45; 95% CI: 0.99–2.11) [10]. Similarly, a meta-analysis carried out with eight prospective studies in the Consortium on Health and Ageing: Network of Cohorts in Europe and the United States (CHANCES) included participants aged 60 years and above and showed that fair and poor/very poor SRH were associated with higher CVD mortality [20]. In our study, the results are consistent with these previous studies, as we observed 54% and 93% higher risk of CVD mortality among those who reported fair and poor/very poor SRH compared to very good/good adjusted for sex and age at 12 years. As in the results of the CHANCES study, the effect for CVD mortality in our study was not statistically significant in multivariable adjusted model [20]. With regard to cancer mortality, few studies have reported associations of poor SRH with higher mortality, but the evidence is very limited [20,25,26]. As in another study carried out in Mediterranean population, we found no significant association with cancer mortality [10]. However, the sample size in our study was small, particularly for the evaluation of the association between SRH and cancer mortality. More research is needed in this direction.

Previous studies have shown inconsistent results by sex for the association between poor SRH and higher all-cause mortality. Some studies have reported an association only in women like in our study [7,27–30] or only in men [25,31], whereas other studies have reported the association in both, men and women [8,32]. Thus, our results are consistent with most of the studies showing an increased risk of all-cause mortality among women who reported their health as poor/very poor, but not among men [7,27–29]. The lack of association in men in our study could be related in part to the much lower sample size for men, as shown in the sensitivity analysis, although it could be due also to the fact that men tend to report less frequently a poor health status than women, even when they have fatal conditions. It may be also possible that women have better health-awareness [7,33].

Regarding the higher mortality related to poor SRH in participants 65–75 years than in those 75 years and older, we have no clear explanation other than the low sample size for the oldest category in our study. Most of the studies have reported an increasing mortality according to age among adults 65 years and older [7,34,35]. We also, explored interaction terms of SRH status with sex and age but they were not significant (data not shown).

We acknowledge the following limitations of our study. Firstly, we explored SRH at baseline with a single-item that may change during follow-up. However, it has been suggested that a single assessment of SRH could be adequate because it remains stable even after major health events [36]. In addition, SRH as a simple measure is widely used in surveys and clinical settings as a screening tool for patients’ health status [1,2,6]. This supports the use of SRH as a measure which is reliable and sufficiently stable over the years [24,36]. Secondly, a high proportion of participants reported the presence of one or more pre-existing chronic diseases at baseline which might have influenced our results. However, we did the analysis adjusting for the main self-reported diseases such as diabetes, hypertension or cholesterol and the effect estimates were basically unchanged. Thirdly, participants were volunteers in cross-sectional studies, and this might have produced some response bias. However, this is unlikely to have biased the estimated associations because SRH did not influence the participation rate and these results were similar to those shown in another study with an older adult population in Spain. Finally, it should be noted that the small sample size could have limited statistical power to detect some associations as statistically significant (e.g CVD or cancer mortality), although the 12 years of follow-up was sufficiently long to detect significant associations with all-cause mortality.

In contrast, our study has several strengths. Firstly, in the present study we had well-defined populations from a Mediterranean area from which we collected high quality information on a considerable number of potential confounders such as sociodemographic characteristics, lifestyles and diet, following standard procedures and validated questionnaires in a personal interview [13–16]. The association between SRH status and mortality was mostly confirmed in sensitivity analyses although the stronger association observed between poor/very poor SRH status and mortality among VNS participants could be due to chance, because of the low number of participants in the poor/very poor SRH category in the VNS (n = 32). Therefore, we were able to obtain adjusted estimates which made confounding less likely. Finally, the information about SRH was obtained at baseline, before the outcome occurred, making differential misclassification more unlikely.

## Conclusion

In summary, this study shows that fair and poor/very poor SRH may be significantly associated with a higher risk of all-cause mortality after 12-years of follow-up in older adults. Our study did not find significant associations with specific causes of death possibly because the statistical power was limited. These results may support the use of SRH as an index to assess mortality risk and to design surveillance preventive strategies in order to prevent mortality in the older adults.

## Supporting information

S1 File (DTA)
